# Prognostic utility of procalcitonin and lactate clearance for in-hospital mortality in sepsis

**DOI:** 10.3389/fmed.2025.1679297

**Published:** 2025-11-20

**Authors:** Razan Diab, Ralphe Bou Chebl, Nour Barmo, Reem Siblini, Maha Makki, Hani Tamim, Gilbert Abou Dagher

**Affiliations:** 1 Department of Emergency Medicine, American University of Beirut Medical Center, Beirut, Lebanon; 2 Department of Internal Medicine, Faculty of Medicine, Clinical Research Institute, American University of Beirut, Beirut, Lebanon; 3 College of Medicine, Alfaisal University, Riyadh, Saudi Arabia

**Keywords:** sepsis, septic shock, procalcitonin, lactate, clearance, mortality

## Abstract

**Background:**

Sepsis remains a significant global health burden and a leading cause of in-hospital mortality. Recent research has focused on the prognostic value of biomarker kinetics, particularly clearance rates and inflammatory markers such as neutrophil-to-lymphocyte (NLR) ratio. This study aimed to compare the utility of procalcitonin and lactate clearance in predicting in-hospital mortality among septic patients and to identify an optimal procalcitonin clearance (PCTc) cut-off to differentiate survivors from non-survivors.

**Methods:**

This was a retrospective cohort study of adult patients who presented with sepsis or septic shock to a tertiary care ED in Lebanon between November 2018 and March 2024. Procalcitonin and lactate readings were recorded along with demographics, comorbidities and therapeutic interventions. PCTc and lactate clearance were calculated as percentage change between the first and second readings, and lactate clearance was considered positive if > 10%. The primary outcome was in-hospital mortality, and secondary outcomes included ED, ICU and hospital length of stay. ROC curve was used to assess prognostic accuracy of biomarkers and derive an optimal PCTc cutoff. Multivariable logistic regression was conducted to evaluate the association of in-hospital mortality with lactate and procalcitonin clearances.

**Results:**

Five hundred seventy-four patients with sepsis and septic shock were included. Mean age was 71.4 ± 16.5 years with male predominance (55.4%). Optimal cutoff for PCTc was found to be 23.1% (94.0% sensitivity, 7.0% specificity). Patients were then stratified based on lactate and procalcitonin clearances above and below the cutoffs to compare baseline parameters, interventions and outcomes. Patients with lactate clearance > 10 had significantly lower rates of chronic kidney disease (*p* = 0.006), congestive heart failure (*p* = 0.02), and chronic obstructive pulmonary disease (*p* = 0.04). Only CRP showed a statistically significant difference with respect to PCTc. Therapeutic interventions were similar in both PCTc groups and lactate clearance groups except for 24-h IV fluid administration (*p* = 0.04). Mortality was significantly associated with lactate clearance > 10 (*p* = 0.045) but not with PCTc (*p* = 0.65). The area under the ROC curve was 0.40 (95% CI: 0.34–0.45, *p* = 0.56) for lactate clearance, 0.39 (95% CI: 0.33–0.45, *p* = 0.56) for PCTc and 0.51 (95% CI: 0.46–0.56, *p* = 0.67) for NLR, with a significant difference among the AUCs (*p* < 0.001). Multivariate analysis showed a borderline significant association of in-hospital mortality with lactate clearance (OR = 0.66, 95% CI 0.42–1.04, *p* = 0.07) but not with procalcitonin clearance (OR = 1.13, 95% CI 0.43–2.95, *p* = 0.81). Vasopressor use was associated with reduced odds of death, while steroid use was independently associated with increased mortality.

**Conclusion:**

Lactate clearance with 10% cutoff is a better predictor of in-hospital mortality in patients presenting to the ED with sepsis or septic shock compared to PCTc. An optimal PCTc cutoff of 23.1% was identified; however, it did not reach statistical significance for survival. Future prospective studies are needed to better define optimal biomarker cutoffs and compare their predictive accuracy for in-hospital mortality.

## Introduction

Sepsis remains a major global health burden and a leading cause of mortality with an estimated 48.9 million incident cases and 11 million sepsis-related deaths annually, accounting for approximately 19.7% of all global deaths (1, 2). Despite advances in early recognition and management, sepsis and septic shock continue to pose diagnostic and prognostic challenges in critical care (2–4). Early identification of high-risk patients and timely assessment of therapeutic response are essential for improving clinical outcomes (5–7). In this context, multiple biomarkers have been widely studied to aid in diagnosis, guide treatment, and monitor response to therapy (8, 9). Yet, no biomarker has been sufficiently useful for accurate diagnosis and outcome prediction, which could potentially guide clinical decision-making (7, 10). This led to exploring biomarker kinetics, serial changes in sepsis biomarkers rather than an isolated value, as a promising tool to identify clinically effective prognosticators of sepsis and septic shock hospitilizations (11).

Lactate is one of the most widely studied biomarkers. As a marker of tissue hypoperfusion and cellular metabolic dysfunction, it has been a well-established component of the sepsis definition, though its clinical utility in prognostication and resuscitation bundles has been deliberately challenged (12–16). While hyperlactatemia typically reflects an imbalance between oxygen delivery and demand with a shift toward anaerobic metabolism, there are other causes for elevated lactate such epinephrine use, malignancies, medication use such metformin and beta agonists (14, 17). This supports the notion that lactate clearance, defined as reduction in serum lactate levels over time, is a more reliable indicator of effective resuscitation in sepsis patients than isolated lactate values (14). Therefore, lactate clearance has emerged as a dynamic biomarker measure for guiding sepsis management and predicting survival. A multicenter study by Ryan et al concluded a value of 10% lactate clearance to be the strongest predictor of mortality in sepsis patients (18). Clearances < 10% were associated with worse outcomes, while more robust clearances were often interpreted as a sign of resolving hypoxia and effective resuscitative efforts (18, 19). Nguyen et al. demonstrated that early lactate clearance in patients with severe sepsis or septic shock is associated with improved morbidity and mortality outcomes (15, 17, 19). Similarly, meta-analyses of randomized controlled trials support lactate clearance-guided therapy, showing significant mortality reduction in septic patients (20–22). Moreover, fluid resuscitation protocols tailored according to lactate clearance have proven both effective and reliable, especially in guiding early interventions while persistently elevated lactate beyond 24 h is associated with mortality rates up to 89% (20–23).

Conversely, procalcitonin, a precursor of the hormone calcitonin, increases in response to severe systemic and bacterial infections, and rise in its levels has been associated with the severity of sepsis and septic shock (24, 25). With appropriate antimicrobial therapy, levels decline making procalcitonin a potentially valuable biomarker of clinical improvement and survival (24, 26). Multiple studies showed that dynamic changes in procalcitonin clearance (PCTc) were associated with survival prediction in sepsis patients admitted to the ICU (26–28).

While both lactate and procalcitonin have individually been shown to correlate with sepsis severity and mortality, there is limited evidence directly comparing their clearance kinetics as predictors of survival. It remains uncertain which biomarker more reliably reflects the resolution of sepsis and better predicts short-term outcomes such as in-hospital mortality. A comparative evaluation with other established prognostic biomarkers in sepsis, such as C-reactive protein (CRP), neutrophil count, and the neutrophil-to-lymphocyte ratio (NLR), is also warranted. Notably, NLR has gained attention as a simple, inexpensive, and readily available indicator of systemic inflammation, reflecting the imbalance between innate and adaptive immunity and demonstrating promising utility in sepsis prognostication (29). Moreover, while the optimal cut-off value of lactate clearance is primarily reported to be 10%, that of procalcitonin remains poorly defined. Therefore, the study aimed to calculate an optimal cut-off value for PCTc that could differentiate survivors from non-survivors, compare PCTc with lactate clearance to identify which better correlates with in-hospital mortality, and evaluate both clearances in relation to inflammatory markers such as CRP and NLR.

## Materials and methods

### Study objective

The objective of this study was to evaluate the prognostic ability of lactate clearance versus procalcitonin clearance to predict in-hospital mortality and to derive a cut off value for procalcitonin clearance.

### Study design

This retrospective cohort study included patients presenting to the Emergency Department (ED) of a tertiary care center in Lebanon with sepsis or septic shock. The study aimed to better define the prognostic utility of procalcitonin and lactate clearance and to compare their performance with each other and with other established biomarkers, including NLR. The study was approved by the Institutional Review Board (IRB) of the American University of Beirut (AUB) with protocol number BIO-2024-0115.

### Patient population

Patient charts were reviewed between November 2021 and March 2024. Adult patients (age ≥ 18 years) diagnosed with sepsis or septic shock with measured procalcitonin and lactate levels in the ED were included. Patients < 18 years were excluded alongside pregnant, transfer or trauma patients, patients presenting with cardiac arrest and those who did not meet the criteria for sepsis or septic shock.

### Data collection

Variables extracted from patient charts included demographics, comorbidities, vital signs and workup done in the ED. Lab tests collected were Complete Blood Count (CBC), Creatinine, Electrolytes, Troponin, INR, Lactate, Procalcitonin, CRP, Albumin and Arterial Blood Gases (ABGs). Other variables retrieved were need for mechanical ventilation, infection site (Lung, Urine, Intravascular Catheter, Gastrointestinal (GI), Skin, Gallbladder, Surgical Site, Blood, Peritoneum, Pyelonephritis), vasopressor use, steroids use, and total fluids received in the ED and at 24 h since admission.

### Outcomes

The primary outcome was mortality with respect to lactate clearance versus procalcitonin clearance, while compared to NLR. Secondary outcomes included ED length of stay (LOS), ICU LOS and hospital LOS.

### Biomarker clearance measurements

Procalcitonin clearance (PCTc) was defined using the following equation (30):


P⁢C⁢T⁢c=(1⁢s⁢t⁢P⁢C⁢T⁢r⁢e⁢a⁢d⁢i⁢n⁢g-2⁢n⁢d⁢P⁢C⁢T⁢r⁢e⁢a⁢d⁢i⁢n⁢g)1⁢s⁢t⁢P⁢C⁢T⁢r⁢e⁢a⁢d⁢i⁢n⁢g⁢x⁢ 100

The first procalcitonin reading is reported if it was taken within the first 24 h. A positive result number indicated clearance of procalcitonin, while a negative value denoted an increase in level of procalcitonin afted initial presentation to the ED.

Lactate clearance is calculated using the following formula (18):


L⁢a⁢c⁢t⁢a⁢t⁢e⁢c⁢l⁢e⁢a⁢r⁢a⁢n⁢c⁢e=(1⁢s⁢t⁢l⁢a⁢c⁢t⁢a⁢t⁢e⁢r⁢e⁢a⁢d⁢i⁢n⁢g-2⁢n⁢d⁢l⁢a⁢c⁢t⁢a⁢t⁢e⁢r⁢e⁢a⁢d⁢i⁢n⁢g)1⁢s⁢t⁢l⁢a⁢c⁢t⁢a⁢t⁢e⁢r⁢e⁢a⁢d⁢i⁢n⁢g⁢x⁢ 100

Lactate clearance cutoff was taken at 10% change between the 1st and 2nd readings reported in our database. Lactate clearance was considered positive if clearance is > 10%, and negative if it is ≤ 10 (18).

To further elaborate on our analysis, we computed NLR from our data and compared its performance to both lactate and procalcitonin clearances in predicting in-hospital mortality.

### Statistical analysis

Analyses were performed using Statistical Package for the Social Sciences (SPSS) version 29 (IBM Corp., Armonk, NY, United States). Data was described using frequencies, percentages, means, standard deviations, medians and interquartile ranges. Shapiro-Wilk test of normality was done to identify highly skewed non-normal variables. Pearson’s chi-square and Student’s *t*-test were used to analyze parametric categorical and continuous variables resepctively. Meanwhile, Mann-Whitney and Fisher’s tests were performed for non-parametric continuous and categorical variables, respectively. Statistical significance was defined as a *p*-value < 0.05 for both tests. Baseline characteristics, lab values, therapy and outcome measures of interest were compared between patients with lactate and procalcitonin clearances below and above the cutoff values. The accuracy of lactate and procalcitonin clearances for predicting in-hospital mortality were assessed using the area under the receiver operating characteristic curve (AUC and ROC) across multiple clinical subgroups including lactate < 2 mmol/L or ≥ 2 mmol/L, albumin < 30 g/L or ≥ 30 g/L, patient subgroups (CKD, Heart failure, malignancy), infection site (lung, urine GI, skin, gallbladder), diabetes and age < 65. An AUC was also derived for NLR and compared to that of lactate clearance and PCTc. Furthermore, the ROC curve was used to determine the optimal cut-off value for PCTc at its maximum sensitivity and specificity, and likelihood ratios were subsequently calculated. Finally, multivariable logistic regression was conducted to study the association of mortality with lactate clearance and procalcitonin clearance after adjusting for clinically and statistically significant variables.

## Results

Our cohort included a total of 574 sepsis and septic shock patients with 295 (51.4%) survivors and 279 (48.6%) non-survivors. The mean age of the cohort was 71.4 ± 16.5 years with a male predominance (55.4%), and no significant difference observed between survivors and non-survivors in age or sex (*p* = 0.49 and *p* = 0.81 respectively). The most common comorbidities were hypertension (HTN) (57.3%), malignancy (49.7%), and diabetes mellitus (DM) (36.1%). Non-survivors were more likely to have congestive heart failure (CHF) (29.4 vs. 21.4%, *p* = 0.03) and malignancy (54.1 vs. 45.4%, *p* = 0.04), while no significant differences were observed for other comorbidities, including chronic kidney disease (CKD), HTN, DM, and chronic obstructive pulmonary disease (COPD). Non-survivors had significantly higher systolic blood pressure (SBP) (111.6 ± 27.3 vs. 105.8 ± 26.5, *p* = 0.01), higher CRP levels (155.6 ± 115.1 mg/L vs. 120.6 ± 98.0 mg/L, *p* < 0.001) and lower hemoglobin levels (10.8 ± 2.3 g/dL vs. 11.3 ± 3.7 g/dL, *p* = 0.04). Lactate clearance was notably lower in non-survivors (−0.23 ± 1.42) compared with survivors (0.18 ± 0.57, *p* < 0.002), whereas neither procalcitonin clearance nor NLR showed any significant difference (*p* = 0.13 and *p* = 0.92 respectively). Vasopressor use was significantly higher in survivors (50.2 vs. 35.5%, *p* < 0.001), while steroids were more frequently used in non-survivors (35.5 vs. 21.7%, *p* < 0.001). Non-survivors also had longer ED, ICU, and hospital stays (*p* = 0.01 and *p* < 0.001 respectively) (Table 1).

**TABLE 1:** Baseline characteristics of patients presenting to the emergency department with sepsis or septic shock.

	Total (*N* = 574)	Survivors (*N* = 295)	Non-survivors (*N* = 279)	*P*-value
Age, mean ± SD	71.4 ± 16.5	70.9 ± 17.8	71.9 ± 14.9	0.49
Male	318 (55.4%)	162 (54.9%)	156 (55.9%)	0.81
Chronic kidney disease	151 (26.3%)	70 (23.7%)	81 (29.0%)	0.15
Hypertension	329 (57.3%)	166 (56.3%)	163 (58.4%)	0.60
Dyslipidemia	148 (25.8%)	82 (27.8%)	66 (23.7%)	0.26
Atrial fibrillation	144 (25.1%)	70 (23.7%)	74 (26.5%)	0.44
Coronary artery disease	168 (29.3%)	92 (31.2%)	76 (27.2%)	0.30
Congestive heart failure	145 (25.3%)	63 (21.4%)	82 (29.4%)	0.03
Malignancy	285 (49.7%)	134 (45.4%)	151 (54.1%)	0.04
History of stroke	39 (6.8%)	19 (6.4%)	20 (7.2%)	0.73
History of vascular disease	126 (22.0%)	61 (20.7%)	65 (23.3%)	0.45
Diabetes mellitus	207 (36.1%)	112 (38.0%)	95 (34.1%)	0.33
Chronic obstructive pulmonary disease	80 (13.9%)	37 (12.5%)	43 (15.4%)	0.32
Systolic blood pressure (mmHg)	108.6 ± 27.1	105.8 ± 26.5	111.6 ± 27.3	0.01
Heart rate (Beats/minute)	101.2 ± 25.1	102.2 ± 25.2	100.2 ± 25.2	0.35
White blood cell count (cu.mm)	13232.7 ± 12897.0	123934.0 ± 11196.9	14119.5 ± 14446.8	0.11
Hemoglobin Hb	11.1 ± 3.1	11.3 ± 3.7	10.8 ± 2.3	0.04
Lactate (mmol/L)	3.5 ± 2.5	3.4 ± 2.4	3.6 ± 2.6	0.40
CRP (mg/L)	137.2 ± 107.7	120.6 ± 98.0	155.6 ± 115.1	<0.001
Procalcitonin (ng/mL)	10.0 ± 23.0	11.4 ± 23.5	8.5 ± 22.4	0.22
Lactate clearance	–0.02 ± 1.10	0.18 ± 0.57	–0.23 ± 1.42	<0.002
Procalcitonin clearance	–27.15 ± 223.78	–7.38 ± 36.67	–47.05 ± 314.14	0.13
Neutrophil to lymphocyte ratio (NLR)	16.97 ± 19.61	17.05 ± 20.13	16.88 ± 19.10	0.92
Creatinine (mg/dL)	1.8 ± 1.4	1.8 ± 1.5	1.7 ± 1.4	0.39
Vasopressor use in the ED	247 (43.0%)	148 (50.2%)	99 (35.5%)	<0.001
Steroids use in the ED	163 (28.4%)	64 (21.7%)	99 (35.5%)	<0.001
Iv fluids in the ED	2441.1 ± 3018.2	2427.5 ± 1605.1	2455.4 ± 4006.4	0.91
Iv fluids in the first 24 h	2508.4 ± 1495.1	2810.5 ± 1375.4	2187.9 ± 1551.8	<0.001
Average length of ED stay (days)	0.9 ± 1.9	0.7 ± 0.8	1.2 ± 2.5	0.01
Average length of ICU stay (days)	9.4 ± 23.5	4.7 ± 6.3	14.4 ± 32.3	<0.001
Average length of hospital stay (days)	18.1 ± 42.7	12.1 ± 10.7	24.4 ± 59.6	<0.001

In this table shows baseline characteristics of sepsis/septic shock patients among survivors and non-survivors.

**FIGURE 1:**
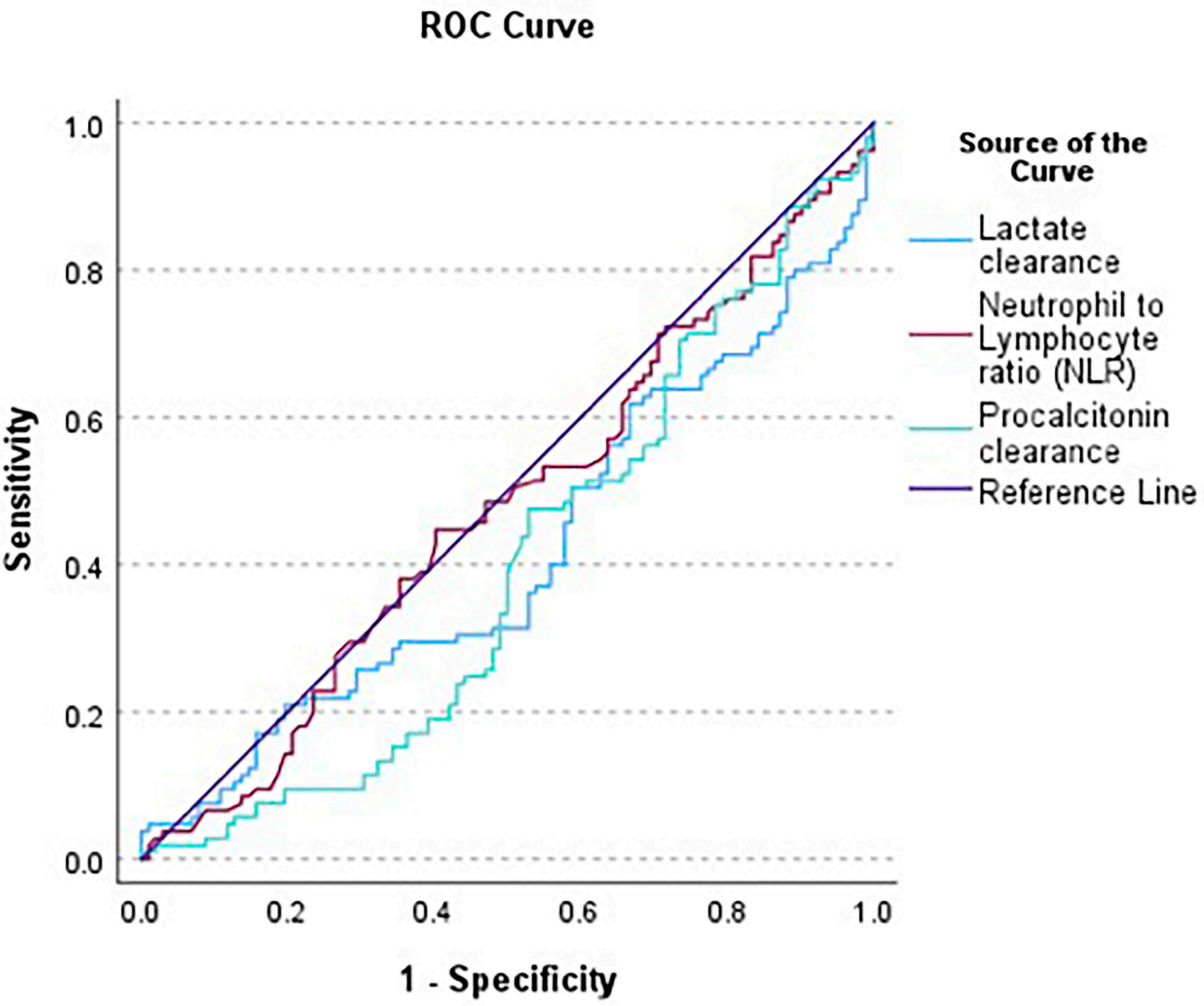
ROC curves of the different biomarkers in relation to mortality.

PCTc cutoff was found to be 23.1%, with a sensitivity of 94.0%, specificity of 7.0%, LR + = 1.01 and LR− = 0.86, using data from our generated sepsis database (Figure 1). Using lactate and procalcitonin clearance cut-offs, we stratified our study sample to compare baseline characteristics, ED parameters and outcomes. Age and sex distributions were similar across groups stratified by lactate clearance (*p* = 0.48 and *p* = 0.96, respectively) and by procalcitonin clearance (*p* = 0.76 and *p* = 0.59, respectively). Patients with lactate clearance > 10 had significantly lower rates of CKD (20.5 vs. 33.1%, *p* = 0.006), CHF (23.6 vs. 34.6%, *p* = 0.02), and COPD (9.4 vs. 16.5%, *p* = 0.04), while other comorbidities were similarly distributed between the 2 lactate clearance groups. However, none of the recorded comorbidities showed significant difference with respect to PCT clearance. Moreover, the most common infection sites in our sample were lung, urinary tract infections and gastrointestinal infections (43.6, 22.6, and 15.2%, respectively). However, infection site was not significantly different in either of the clearance groups (lactate clearance, *p* = 0.29; PCTc, *p* = 0.98) (Table 2).

**TABLE 2:** Characteristics of patients with sepsis or septic shock stratified by lactate and procalcitonin clearances.

	Total (*N* = 574)	Lactate clearance ≤ 10 (*N* = 133)	Lactate clearance > 10 (*N* = 254)	*P*-value	PCT clearance ≤ 23.11 (*N* = 20)	PCT clearance > 23.11 (*N* = 279)	*P*-value
Age, years	71.4 ± 16.5	71.96 ± 15.77	70.68 ± 17.73	0.48	73.60 ± 18.37	72.47 ± 15.66	0.76
Male	318 (55.4%)	74 (55.6%)	142 (55.9%)	0.96	12 (60.0%)	150 (53.8%)	0.59
**Comorbidities, %**							
CKD	151 (26.3%)	44 (33.1%)	52 (20.5%)	0.006	8 (40.0%)	73 (26.2%)	0.18
HTN	329 (57.3%)	76 (57.1%)	142 (55.9%)	0.82	11 (55.0%)	165 (59.1%)	0.72
Dyslipidemia	148 (25.8%)	34 (25.6%)	65 (25.6%)	0.99	7 (35.0%)	73 (26.2%)	0.39
Afib	144 (25.1%)	32 (24.1%)	66 (26.0%)	0.68	6 (30.0%)	83 (29.7%)	0.98
CAD	168 (29.3%)	36 (27.1%)	72 (28.3%)	0.79	6 (30.0%)	83 (29.7%)	0.98
CHF	145 (25.3%)	46 (34.6%)	60 (23.6%)	0.02	6 (30.0%)	79 (28.3%)	0.87
Malignancy	285 (49.7%)	63 (47.4%)	124 (48.8%)	0.79	6 (30.0%)	136 (48.7%)	0.105
History of stroke	39 (6.8%)	11 (8.3%)	19 (7.5%)	0.78	3 (15.0%)	22 (7.9%)	0.27
History of vascular disease	126 (22.0%)	34 (25.6%)	65 (25.6%)	0.99	3 (15.0%)	67 (24.0%)	0.36
DM	207 (36.1%)	49 (36.8%)	98 (38.6%)	0.74	6 (30.0%)	103 (36.9%)	0.54
COPD	80 (13.9%)	22 (16.5%)	24 (9.4%)	0.04	2 (10.0%)	43 (15.4%)	0.51
**Site of infection, %**							
Lung	250 (43.6%)	63 (47.4%)	100 (39.4%)	0.29	11 (55.0%)	148 (53.0%)	0.98
Urine	130 (22.6%)	29 (21.8%)	58 (22.8%)		4 (20.0%)	57 (20.4%)	
Intravascular cath	9 (1.6%)	2 (1.5%)	3 (1.2%)		0 (0.0%)	2 (0.7%)	
Gastrointestinal	87 (15.2%)	19 (14.3%)	44 (17.3%)		2 (10.0%)	29 (10.4%)	
Skin	20 (3.5%)	4 (3.0%)	8 (3.1%)		0 (0.0%)	11 (3.9%)	
Gallbladder	27 (4.7%)	4 (3.0%)	19 (7.5%)		1 (5.0%)	11 (3.9%)	
Surgical site	7 (1.2%)	1 (0.8%)	4 (1.6%)		0 (0.0%)	3 (1.1%)	
Blood	19 (3.3%)	3 (2.3%)	10 (3.9%)		2 (10.0%)	9 (3.2%)	
Pyelonephritis	5 (0.9%)	1 (0.8%)	3 (1.2%)		0 (0.0%)	1 (0.4%)	

PCT, Procalcitonin; CKD, Chronic Kidney Disease; HTN, Hypertension; Afib, Atrial fibrillation; CAD, Coronary Artery Disease; CHF, Congestive Heart Failure; DM, Diabetes Mellitus; COPD, Chronic Obstructive Pulmonary Disease. In this table shows characteristics of sepsis/septic shock patients compared between and within lactate and procalcitonin clearance groups.

None of the reported vitals reached statistical significance in either the lactate clearance or the PCTc counterpart. In terms of laboratory values, there were no significant differences in relation to lactate clearance, while only CRP showed a statistically significant difference with respect to PCTc (*p* < 0.001) (Table 3).

**TABLE 3:** Emergency department (ED) presentation parameters and laboratory values.

	Total (*N* = 574)	Lactate clearance ≤ 10 (*N* = 133)	Lactate clearance > 10 (*N* = 254)	*P*-value	PCT clearance ≤ 23.11 (*N* = 20)	PCT clearance > 23.11 (*N* = 279)	*p*-value
**Vital signs**							
SBP (mmHg)	108.6 ± 27.1	108.59 ± 26.54	107.96 ± 28.00	0.83	118.90 ± 30.07	109.09 ± 26.71	0.12
DBP (mmHg)	66.0 ± 18.9	65.77 ± 19.17	65.88 ± 18.68	0.96	72.25 ± 18.38	66.53 ± 19.52	0.205
HR (bpm)	101.2 ± 25.1	102.62 ± 22.20	102.83 ± 25.79	0.94	109.40 ± 20.18	100.35 ± 24.54	0.108
O2sat (%) *	97.0 (6.0)	96.0 (6.0)	97.0 (6.0)	0.21	96.0 (4.0)	96.0 (7.0)	0.95
Temperature (C)	37.3 ± 1.2	37.24 ± 1.24	37.33 ± 1.17	0.48	37.43 ±	37.28 ±	0.59
Respiratory rate (Breaths/min)*	20.0 (6.0)	20.0 (4.0)	20.0 (6.0)	0.14	20.0 (7.0)	20.0 (6.0)	0.97
**Lab values**							
White blood cell count (cu.mm)*	11,050 (10,025)	10,400 (10,950)	11,250 (9,725)	0.20	12,500 (13,465)	11,100 (9400)	0.53
Hemoglobin Hb	11.1 ± 3.1	11.23 ± 4.45	11.13 ± 2.66	0.78	11.97 ± 2.10	11.05 ± 3.17	0.207
Lactate (mmol/L)*	2.79 (2.58)	2.51 (2.11)	3.76 (3.17)	<0.001	2.61 (1.08)	2.74 (2.43)	0.99
CRP (mg/L)*	111.3 (154.3)	98.8 (127.4)	124.8 (176.4)	0.17	36.35 (77.65)	116.20 (138.35)	<0.001
Albumin (g/L)	27.8 ± 6.5	27.77 ± 6.32	27.53 ± 6.70	0.76	30.47 ± 7.40	27.78 ± 5.70	0.07
Procalcitonin*(ng/mL)	1.02 (5.65)	1.08 (6.0)	1.67 (8.6)	0.32	0.26 (0.69)	0.97 (5.72)	<0.001
Creatinine Cr (mg/dL)	1.8 ± 1.4	1.81 ± 1.54	1.73 ± 1.26	0.59	1.63 ± 0.99	1.73 ± 1.54	0.78
Bicarbonate (mmol/L)	21.9 ± 5.6	21.58 ± 5.80	21.21 ± 5.30	0.53	21.75 ± 4.29	22.72 ± 5.94	0.47
Troponin*	0.054 (0.07)	0.054 (0.17)	0.053 (0.07)	0.14	0.05 (0.07)	0.048 (0.07)	0.96
pH (ABG)	7.4 ± 0.1	7.38 ± 0.10	7.40 ± 0.10	0.13	7.32 ± 0.15	7.39 ± 0.11	0.08
pCO_2_ (ABG)	36.4 ± 12.8	35.38 ± 12.86	34.20 ± 10.22	0.46	42.11 ± 22.87	37.95 ± 12.73	0.50

*Median and IQR were reported for highly-skewed non-parametric variables. PCT, Procalcitonin; SBP, Systolic Blood Pressure; DBP, Diastolic Blood Pressure; HR, Heart rate; O2sat, Oxygen saturation; CRP, C-reactive Protein. In this table shows ED parameters and lab values of sepsis/septic shock patients compared between and within lactate and procalcitonin clearance groups.

Both vasopressor use and steroid use were not significantly different between the 2 lactate clearance groups (*p* = 0.36 and *p* = 0.31, respectively). Similarly, they did not differ with respect to PCTc (*p* = 0.19 and *p* = 0.29, respectively). On the other hand, patients with high lactate clearance > 10 received more IV fluids in the first 24 h of ED presentation (*p* = 0.04) compared to patients with a lower lactate clearance. In the PCTc arm, fluid resuscitation did not show a statistically significant difference between the 2 groups. Moreover, ED, ICU, and hospital lengths of stay did not differ significantly with either lactate clearance or procalcitonin clearance. Finally, 56.4% of patients with lactate clearance ≤ 10 died within their hospital stay compared to 45.7% with lactate clearance > 10, with a significant association between lactate clearance and survival (*p* = 0.045). Meanwhile, no significant difference in mortality rates was shown between PCTc groups below and above the cutoff (45.0 vs. 50.2%, *p* = 0.65) (Table 4).

**TABLE 4:** Therapeutic interventions, outcomes and mortality in lactate and procalcitonin clearance groups.

	Total (*N* = 574)	Lactate clearance ≤ 10 (*N* = 133)	Lactate clearance > 10 (*N* = 254)	*P*-value	PCT clearance ≤ 23.11 (*N* = 20)	PCT clearance > 23.11 (*N* = 279)	*p*-value
**Therapy**							
Vasopressor use in ED	247 (43.0%)	63 (47.4%)	108 (42.5%)	0.36	11 (55.0%)	112 (40.1%)	0.19
Steroids use in ED	163 (28.4%)	41 (30.8%)	66 (26.0%)	0.31	4 (20.0%)	87 (31.2%)	0.29
Iv fluids in ED*	1,982 (2,081)	1,700 (2,186)	2,140 (2,067)	0.07	2,310 (2,384)	1,700 (2,106)	0.90
Iv fluids in 1st 24_h	2508.4 ± 1495.1	2440.77 ± 1590.43	2770.18 ± 1421.05	0.04	2955.26 ± 1411.71	2393.36 ± 1478.75	0.10
Mechanical ventilation	298 (51.9%)	69 (51.9%)	128 (50.4%)	0.78	11 (55.0%)	162 (58.1%)	0.79
**Outcomes**							
ED LOS (days)*	0.49 (0.67)	0.50 (0.71)	0.50 (0.69)	0.54	0.54 (1.22)	0.46 (0.71)	0.79
ICU LOS (days)*	3.52 (8.50)	5.46 (11.62)	3.00 (7.25)	0.14	3.00 (8.38)	5.71 (10.88)	0.85
Hospital LOS (days)*	10.35 (14.16)	12.73 (16.29)	9.87 (14.83)	0.21	8.06 (13.28)	12.25 (15.16)	0.97
**Mortality**							
Survivors	295 (51.4%)	58 (43.6%)	138 (54.3%)	0.045	11 (55.0%)	139 (49.8%)	0.65
Non-survivors	279 (48.6%)	75 (56.4%)	116 (45.7%)		9 (45.0%)	140 (50.2%)	

*Median and IQR were reported for highly-skewed non-parametric variables. ED, Emergency Department; ICU, Intensive Care Unit; LOS, Length of Stay; PCT, Procalcitonin. In this table shows therapeutic interventions, outcomes and mortality of sepsis/septic shock patients with respect to lactate and procalcitonin clearances above and below their respective cutoff values.

Both lactate clearance and PCTc demonstrated poor discriminatory ability for in-hospital mortality, with overall AUCs of 0.40 (95% CI: 0.34–0.45) and 0.39 (95% CI: 0.33–0.45), respectively (*p* = 0.56). Subgroup analyses showed similarly low predictive performance across different lactate and albumin levels, with AUCs below 0.5 and no significant differences between groups. Among comorbidities, AUCs remained low, though in patients with chronic kidney disease, lactate clearance reached an AUC of 0.43 compared to 0.33 for PCTc, with a near-significant *p*-value of 0.054. In malignancy, lactate clearance reached an AUC of 0.47, but the comparison remained non-significant between groups (*p* = 0.59). Regarding infection sites, skin infections had a slightly higher AUC of 0.53 for lactate clearance, but without statistical significance (*p* = 1.00); other infection sites showed low AUCs. In patients under 65 years, PCTc reached an AUC of 0.56 without significant difference (*p* = 0.64). For sepsis and septic shock patients, lactate clearance AUCs were 0.36 and 0.42, respectively (*p* = 0.56), and procalcitonin clearance AUCs were 0.46 and 0.38, respectively (*p* = 0.19) (Table 5). The AUC of NLR was similarly weak, with a value of 0.51 (95% CI: 0.46–0.56) and a *p*-value of 0.67 for mortality. When comparing all three parameters, a statistically significant difference was observed among the AUCs (*p* < 0.001). Pairwise comparisons also showed significant differences between NLR and lactate clearance (*p* = 0.005) and between NLR and PCT clearance (*p* = 0.04) (Figure 1).

**TABLE 5:** AUCs of lactate and procalcitonin clearances within multiple subgroups.

	AUC for in-hospital mortality (95% CI)		
	Lactate clearance	PCT clearance	*P*-value
Overall	0.40 (0.34–0.45)	0.39 (0.33–0.45)	0.56
**Lactate levels**			
Lactate <2 mmol/L	0.44 (0.32–0.56)	0.41 (0.29–0.52)	0.31
Lactate ≥ 2 mmol/L	0.40 (0.34–0.47)	0.38 (0.30–0.46)	0.48
**Albumin levels**			
Albumin <30 g/L	0.39 (0.32–0.47)	0.43 (0.33–0.52)	0.54
Albumin ≥ 30 g/L	0.39 (0.29–0.49)	0.32 (0.22–0.43)	0.75
Chronic kidney disease	0.43 (0.32–0.55)	0.33 (0.21–0.45)	0.054
Malignancy	0.47 (0.38–0.55)	0.43 (0.33–0.54)	0.59
Heart failure	0.39 (0.28–0.50)	0.39 (0.26–0.51)	0.43
**Infection site**			
Lung	0.40 (0.31–0.49)	0.41 (0.32–0.50)	0.31
Urine	0.42 (0.30–0.55)	0.39 (0.24–0.53)	0.58
Gastrointestinal	0.45 (0.30–0.59)	0.38 (0.18–0.58)	0.90
Skin	0.53 (0.14–0.93)	0.46 (0.10–0.83)	1.00
Gall bladder	0.34 (0.08–0.61)	0.50 (0.15–0.85)	0.67
Diabetes	0.36 (0.27–0.45)	0.35 (0.25–0.45)	0.79
**Age**			
< 65	0.43 (0.33–0.54)	0.56 (0.42–0.69)	0.64
**Diagnosis**			
Sepsis	0.36 (0.19–0.54)	0.46 (0.29–0.62)	0.56
Septic shock	0.42 (0.35–0.48)	0.38 (0.31–0.45)	0.19

PCT, Procalcitonin. In this table shows AUCs for in-hospital mortality for lactate and procalcitonin clearance in overall sample of sepsis/septic shock patients and within individual subgroups.

Finally, in the multivariate analysis and after adjusting for potential confounders, lactate clearance showed a borderline association with in-hospital mortality (OR = 0.66, 95% CI 0.42–1.04, *p* = 0.07), while no association was established with procalcitonin clearance (OR = 1.13, 95% CI 0.43–2.95, *p* = 0.81). In both the lactate and procalcitonin models, vasopressor use was associated with reduced odds of death (OR = 0.46, 95% CI 0.29–0.71, *p* < 0.001; OR = 0.54, 95% CI 0.33–0.90, *p* = 0.02, respectively), while steroid use was independently associated with increased mortality (OR = 1.93, 95% CI 1.17–3.17, *p* = 0.01; OR = 2.02, 95% CI 1.19–3.43, *p* = 0.009, respectively) (Table 6).

**TABLE 6:** Odds ratio of mortality in patients with sepsis and septic shock with respect to lactate and procalcitonin clearance.

	Mortality (reference: No)			
	95%CI			
	OR	Lower	Upper	*P*-value
Lactate clearance	0.66	0.42	1.04	0.07
Vasopressors use	0.46	0.29	0.71	<0.001
Steroids use	1.93	1.17	3.17	0.01
Variables included in the model were: Imposed: Lactate clearance (reference: ≤ 10) Stepwise: Gender (reference: male); CKD; HTN; Dyslipidemia; Afib; CAD; CHF; Malignancy; History of stroke; History of Vascular Disease; DM; COPD; SBP; DBP; HR; O2sat; Temperature; Cr; Vasopressors; Steroids; Iv fluids in the ED				
	**95%CI**			
	**OR**	**Lower**	**Upper**	***P*-value**
Procalcitonin clearance	1.13	0.43	2.95	0.81
Vasopressors use	0.54	0.33	0.90	0.02
Steroids use	2.02	1.19	3.43	0.009
Variables included in the model were: Imposed: Procalcitonin clearance (reference: ≤ –23.11) Stepwise: Gender (reference: male); CKD; HTN; Dyslipidemia; Afib; CAD; CHF; Malignancy; History of stroke; History of Vascular Disease; DM; COPD; SBP; DBP; HR; O2sat; Temperature; Cr; Vasopressors; Steroids; Iv fluids in the ED.				

OR, odds ratio; CI, confidence interval. In this table shows multivariable logistic regression which demonstrated borderline significance between mortality and lactate clearance compared to no significant association with procalcitonin clearance after adjusting for all possible clinically or statistically significant variables.

## Discussion

Our study looked at a cohort of patients with sepsis and septic shock to evaluate the prognostic ability of procalcitonin and lactate clearances for in-hospital mortality. We found a PCTc of 23.1% (sensitivity, 94.0%; specificity 7.0%) to be the optimal cut-off to differentiate survivors from non-survivors. Other studies reported cutoffs ranging from 30 to 70% at different time intervals (24, 31–33). Results revealed 50.2% mortality with higher PCTc and 56.4% mortality with lower lactate clearance. However, only lactate clearance was significantly associated with in-hospital mortality (*p* = 0.045).

Our findings align with prior literature in affirming the prognostic utility of lactate clearance in sepsis. Several studies and meta-analyses have reported strong associations between higher lactate clearance and reduced mortality in sepsis patients (18–21, 23, 34). In particular, a 10% lactate clearance cutoff was shown to be significantly associated with better outcomes. This corroborates prior evidence showing similar survival prediction with a 10% lactate clearance cut-off (18, 19). A study on neonatal sepsis showed higher mortality among septic neonates with < 10% lactate clearance (35). Conversely, Lee et al. identified an optimal lactate clearance cut-off of 24.4% for predicting 30-day mortality and concluded that 6-h lactate levels were superior to 6-h lactate clearance in mortality prediction, a result reproduced by Ryoo et al in their study on lactate clearance in patients with septic shock (36, 37). Another study also found that lactate clearance < 20% was associated with increased in-hospital mortality while that < 10% was not (38). Despite this present controversy on its optimal cutoff, lactate clearance remains a reliable predictor of sepsis-induced mortality.

Procalcitonin clearance showed no significant association with survival of our sepsis and septic shock cohort (*p* = 0.65). A lack of association was also demonstrated in the study by Karlsson et al., which found that a procalcitonin decrease of more than 50% within 72 h was not independently associated with in-hospital mortality (39). In pediatric sepsis, one study found that 48-h PCT did not predict early clinical stability (40), contrasting with another showing reduced 24-h PCT clearance independently associated with higher mortality (41). Thus, evidence on reliability of PCTc as a predictor of mortality remains conflicting. Several studies supported it as a prognosticator of severe sepsis and septic shock (24, 26–28, 31, 32). A 2017 multicenter study concluded that PCT kinetics in the first 4 days were predictive for survival of patients diagnosed with severe sepsis or septic shock (42). Additionally, two systematic review and meta-analysis revealed a significant association between procalcitonin non-clearance and mortality despite significant heterogeneity across included studies (43, 44). Notably, a recent study explored a prognostic advantage of combining PCTc with blood parameters, such as white cell count, platelet count and CRP, for predicting mortality in cancer patients with sepsis (45). In our study, some patients did not have a second procalcitonin level recorded within the first 24 h of admission, resulting in a small sample size. This may have biased the analysis toward a smaller subset of patients who had two documented readings and were thus included in the final analysis.

Our results also showed patients with lower lactate clearance but not PCTc were more likely to have comorbidities including CKD and CHF. Similarly, non-survivors exhibited a higher overall comorbidity burden, with increased rates of CHF and malignancy. Multiple studies highlighted that patients admitted for sepsis or septic shock with concomitant malignancy or heart failure had higher rates of in-hospital mortality (46–49). We believe this reflects an intrinsic relationship between having comorbidities, the ability to clear lactate and mortality. Interestingly, a study by Thomas-Rüddel et al. reported that mortality attributable to sepsis alone ranged from 6 to 12%, whereas mortality involving both sepsis and underlying comorbidities rose to 54–76% (50).

CRP levels were significantly higher in patients with high PCTc, similar to prior findings (51) Serial decrease in PCT remains a better predictor of survival compared to CRP, however, this could hint to the potential benefit of combining both parameters for a more accurate risk assessment and outcome prediction (45, 51).

The neutrophil-to-lymphocyte ratio (NLR) has emerged as an inexpensive and readily obtainable biomarker for prognostication in infectious clinical settings, including sepsis, pneumonia, and COVID-19 (29). Several studies have shown that elevated NLR strongly predicts 30-day mortality in acutely ill elderly patients and in those with community-acquired pneumonia (CAP), a common precursor of sepsis, highlighting its association with disease severity and overall mortality (52, 53). Notably, NLR has been shown to outperform traditional prognostic markers such as CURB-65, CRP, and individual leukocyte counts (52, 54). Furthermore, a meta-analysis including more than 11,000 patients with sepsis reported that higher baseline NLR values were significantly associated with worse outcomes (hazard ratio ≈ 1.75; 95% CI 1.56–1.97) (55).

Despite showing only modest prognostic value overall, NLR outperformed lactate and procalcitonin clearances in our analysis, achieving the largest area under the ROC curve and reinforcing its potential as a powerful biomarker for sepsis prognostication. The discrepancy in results can be potentially explained by unmeasured confounding factors and the heterogeneity of our patient population with variability in sepsis presentation across patients. Additionally, the relatively limited performance of NLR could be partly due to the absence of serial measurements. These limitations underscore the need for future studies to assess NLR as a dynamic marker, as illustrated by Lee H. et al., showing that incremental changes in NLR significantly predicted 30-day mortality in patients with CAP (56).

Combining dynamic biomarkers with established scoring systems such as SOFA, APACHE II, and qSOFA enhances prognostic accuracy in sepsis and septic shock. For example, Li et al. demonstrated that combination of NLR with SOFA outperformed each individually and exhibited predictive performance comparable to APACHE II, while models incorporating lactate or PCT clearance with qSOFA or SOFA were shown to outperform either measure alone in multiple studies, improving early identification of high-risk patients (57–61). By leveraging both laboratory and clinical parameters, these multimodal approaches offer a robust tool for mortality prediction and risk stratification. Future studies should explore combining lactate and PCT clearance with NLR and other dynamic scores to further enhance predictive performance and guide individualized care.

Finally, our results indicated that vasopressor use was significantly higher among survivors and independently associated with decreased odds of in-hospital death. We find this result plausible because rapid restoration of mean arterial pressure (MAP) improves organ perfusion and reduces the need for excessive fluid resuscitation, thereby lowering the risk of fluid overload and improving outcomes. Growing evidence further supports the prognostic benefit of early vasopressor use in septic patients. For example, a randomized phase II trial in patients with sepsis-associated hypotension demonstrated significantly improved shock control at 6 h with early low-dose norepinephrine, though without a significant mortality difference (62). Subsequent systematic reviews and meta-analyses indicated that vasopressor initiation within 1–6 h of shock onset is associated with lower short-term mortality, faster attainment of target MAP and reduced fluid volume (63, 64). Therefore, our findings align with evidence suggesting improved outcomes with earlier, protocolized vasopressor use, yet prospective studies are warranted to further evaluate this intervention, given the inherent confounding bias of retrospective analyses. Conversely, steroid use was significantly higher among non-survivors and associated with increased odds of mortality. While this could likely reflect a confounding state with corticosteroids often reserved for patients with refractory shock or a severe disease course, a systemic review of 61 RCTs demonstrated a slightly reduced 28-day mortality with steroid use, though it had little to no effect on long-term mortality (65). Other trials have demonstrated faster shock resolution with steroid use but did not show a clear mortality benefit, with potential adverse effects contributing to worse outcomes in high-risk patients (66, 67).

Importantly, our study adds to the literature by directly comparing lactate and procalcitonin clearances in a real-world ED population of sepsis and septic shock patients. Lactate clearance appeared to be a better predictor for in-hospital mortality than procalcitonin clearance. Moreover, our subgroup analyses across comorbidities, age groups, and infection sources provide further insight into the predictive performance of both biomarkers, helping to address the gap in understanding which biomarker perform better across heterogeneous sepsis presentations. By confirming lactate clearance threshold that better correlates with mortality and evaluating its performance in key clinical subgroups, our study contributes practical data to help refine biomarker-based resuscitation strategies.

The study presents several important limitations. First, the timing of lactate measurement was not standardized and patients without serial biomarker measurements were excluded, which may have introduced variability and selection bias. Second, there is a risk of survivor bias, as patients who died early may not have had the opportunity to demonstrate clearance, possibly overestimating the association between low clearance and mortality. Missing data on important parameters such as PaO_2_, FiO_2_ and platelets limited our ability to compare clearances with established biomarkers to determine the better mortality predictors and strengthen our analysis. Additionally, the retrospective, single-center design and exclusion of patients lacking Sepsis-3 criteria data (245 of the initial 819 patient dataset) may limit generalizability and applicability to less severe sepsis, while the moderate sample size could have reduced statistical power for some subgroup analyses. Future prospective, multicenter studies with standardized biomarker timing and validated clinical severity scoring are warranted to confirm and expand upon these results.

## Conclusion

When comparing procalcitonin and lactate clearances, the latter appears to be a more reliable predictor of mortality in patients presenting to the emergency department with sepsis or septic shock. While an optimal procalcitonin clearance cutoff of 23.1% was identified for survival, it did not reach statistical significance as a predictor of mortality. In contrast, a lactate clearance of 10% continues to demonstrate prognostic value, despite some controversy in the literature. Our findings also support a prognostic potential for NLR. Future studies are needed to better define optimal cutoffs for dynamic biomarkers and investigate models that integrate biomarkers with clinical scoring systems to improve risk stratification and mortality prediction in sepsis and septic shock.

## Data Availability

The raw data supporting the conclusions of this article will be made available by the authors, without undue reservation.
